# Real-World Effectiveness, Tolerability, and Safety of Dolutegravir/Lamivudine in Korea

**DOI:** 10.3390/v14112558

**Published:** 2022-11-18

**Authors:** Ki Hyun Lee, Jinnam Kim, Jung Ah Lee, Chang Hyup Kim, Jin Young Ahn, Su Jin Jeong, Nam Su Ku, Jun Yong Choi, Joon-Sup Yeom, Young Goo Song, Jung Ho Kim

**Affiliations:** Department of Internal Medicine and AIDS Research Institute, Yonsei University College of Medicine, Seoul 03722, Republic of Korea

**Keywords:** dolutegravir/lamivudine, HIV infection, Asia, effectiveness, safety

## Abstract

Most studies on the real-world effectiveness and safety of dolutegravir/lamivudine (DTG/3TC) have been conducted in Western countries, and Asian reports are lacking. We evaluated the effectiveness and safety of DTG/3TC in Korean adult people living with HIV (PLWH). This retrospective study was conducted from July 2020 to July 2022 at a tertiary hospital in Korea. Those who were followed up for more than 12 months were included. We analyzed the baseline characteristics, effectiveness, resistant profiles, body weights, metabolic parameters, and safety of DTG/3TC treatment in 151 PLWH, dividing them into the treatment-naïve group and the switching group. The median DTG/3TC treatment durations in the treatment-naïve and switching groups were 507.5 and 525.0 days. In the treatment-naïve group, the viral RNA titer was undetectable at 6 and 12 months in 95% of patients. In the switching group, virologic suppression was well-maintained. Meanwhile, the creatinine levels were slightly elevated in both groups compared to baseline. Five participants complained of mild side effects, such as indigestion, constipation, diarrhea, and fatigue. However, no patient stopped treatment during the follow-up period. Since there was no virological failure or serious complications observed in this study, DTG/3TC may be a good treatment option for PLWH in Korea.

## 1. Introduction

Since the discovery of the human immunodeficiency virus (HIV) in 1983, highly active antiretroviral therapy (HAART) has been developed [1]. Because of these therapies, HIV has become a chronic disease and the patients are referred to as people living with HIV (PLWH) [2]. As ART is now a lifelong treatment, the duration of ART is considerable. Therefore, the demand for two-drug regimens (2DRs) is increasing to minimize drug exposure, reduce drug–drug interactions, and alleviate side effects.

Dolutegravir (DTG)-based 2DRs are attractive because of the following advantages: a high barrier to resistance, low risk of drug–drug interactions, and daily dosing schedule [3,4]. For these reasons, studies on the effects, efficacy, and side effects of 2DRs using DTG as an anchor are rapidly progressing [5,6,7]. In the GEMINI phase 3 clinical trial on treatment-naïve patients, a non-inferior efficacy and similar tolerability of DTG/lamivudine (DTG/3TC) compared to a three-drug regimen (3DR) was observed during a 48-week observation period; therefore, it was recommended as an initial therapy in ART-naïve patients [7]. In another large study, TANGO, DTG/3TC was introduced as a good method for drug simplification because it maintains virologic suppression well and does not show ART resistance compared to tenofovir alafenamide (TAF)-based 3DR or four-drug regimens [6].

In the Republic of Korea, DTG/3TC was approved by the Ministry of Food and Drug Safety in early 2020. It is considered a good treatment option for treatment-naïve PLWH or virologically suppressed PLWH with other ARTs. According to the HIV clinical practice guideline recommendation in Korea (revised in 2021), it corresponds to recommendation level 1A for treatment-naïve PLWH with HIV RNA titers less than 500,000 copies/mL [8]. However, most studies on the efficacy and safety of this drug have been conducted in Western countries, and real-world Asian reports are insufficient. In addition, the pharmacokinetics and pharmacodynamics of some ARTs may vary slightly by race [9]; therefore, it may be difficult to apply the results of studies (regarding treatment effects and side effects) conducted in Western countries to Asian countries, such as Korea. Therefore, this study aimed to evaluate the safety and effectiveness of DTG/3TC in Korea.

## 2. Materials and Methods

### 2.1. Study Design and Population

We performed a single-center retrospective observational study at a tertiary hospital with 2400 beds in South Korea. We analyzed all PLWH aged > 19 years who were treated with DTG/3TC, including the treatment-naïve and those who switched from other ARTs to DTG/3TC, from July 2020 to July 2022. Those who were followed up for more than 12 months were included, and the window period for laboratory tests was 1 month. We investigated the baseline characteristics, clinical outcomes, adverse events, and antiretroviral resistance test results for the previously treated and treatment-naïve groups. This study was approved by the institutional review board (IRB) of Severance Hospital. The IRB waived the requirement for written consent from patients, as this study followed a retrospective design (IRB authorization number: 4-2021-1311).

### 2.2. Study Outcomes and Covariates

We measured the initial viral load, CD4+ T cell count, and CD8+ T cell count at baseline, 6 and 12 months after treatment with DTG/3TC was started to evaluate the treatment effects, and we reviewed the side effects in each patient. Other important blood tests, such as lipid profile, renal function, liver function, and blood glucose level assessments, as well as body weight determination, were performed at baseline before treatment and after 6 and 12 months of treatment. The HIV diagnosis and AIDS definitions followed the definition of the World Health Organization (WHO). Viral suppression was defined as an undetectable viral load, equal to or less than 50 copies/mL. Low-level viremia was defined as one or more viral load results that are detectable (more than 50 copies/mL) but equal to or less than 1000 copies/mL. Virological failure was defined as a persistently detectable viral load exceeding 1000 copies/mL after at least 6 months of undergoing ART [10]. We evaluated safety based on the observation of serious side effects.

### 2.3. Virological Analyses

All virological analyses were performed at the Virology Unit laboratory and Department of Diagnostic Laboratory Medicine at Severance Hospital. HIV RNA was quantified by PCR using a VIDAS (bioMerieux SA, Marcy l’Etoile, France), which gives three possible outputs: (i) a quantitative result for HIV RNA values of ≥20 copies/mL; (ii) a semi-quantitative result (detectable below 20 copies/mL) when HIV RNA is detectable but not precisely quantifiable; and (iii) a qualitative result (‘undetectable’) when no signal can be detected. HIV RNA was defined as being undetectable when no signal could be detected (‘target not detected’). Genotyping drug resistance tests were performed using the ViroSeq HIV-1 Genotyping System 2.0 (Abbott Molecular, Des Plaines, IL, USA), and antiretroviral resistance mutations were identified using the Stanford HIV Drug Resistance Database 9.0 “https://hivdb.stanford.edu/page/algorithm-updates/ (accessed on 1 March 2021)”.

### 2.4. Statistical Analysis

All statistical analyses were performed using SPSS Statistics for Windows version 26.0 (IBM, Armonk, NY, USA). Descriptive statistics were used to summarize the patient demographics. When the values were normally distributed, the mean and standard deviation(SD) were used, and when the values were not normally distributed, the median and interquartile range(IQR) were used for comparison. Categorical variables were compared between the treatment-naïve and switching groups using the χ^2^ test or Fisher’s exact test. Continuous variables were compared between the treatment-naïve and switching groups using the unpaired t-test for normally distributed data or the Mann–Whitney U test for non-normally distributed data. The paired T-test, Wilcoxon signed-rank test and repeated measure ANOVA was used to determine the effects and side effects before and after the treatment. Statistical significance in the multivariate logistic regression analysis was defined as *p* < 0.05.

## 3. Results

### 3.1. Demographic and Clinical Characteristics

**Figure 1:**
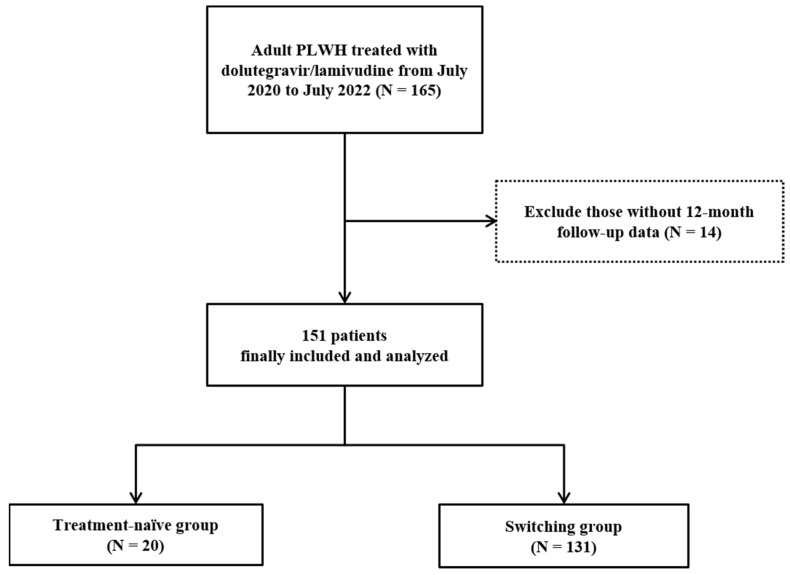
Flow chart for the participant selection method used in this study.

Among the 165 PLWH administered DTG/3TC, 151 patients were enrolled in the study (Figure 1). As shown in Table 1, the baseline characteristics of these 151 PLWH, who were divided into the treatment-naïve group and previously treated with ART group, or switching group, were analyzed. In both groups, most of the patients were male, and the median age in the treatment-naïve group was 28.0 years, which was lower than that of the switching group (45.0 years). The median DTG/3TC treatment duration was 507.5 days in the treatment-naïve group and 525.0 days in the switching group. Sexual orientation only targeted those who responded, with men having sex with men (MSM) being the most common (46.2% and 53.7% respectively).

Of the 131 people in the switching group, 110 (84.0%) were changed to DTG/3TC for drug simplification, and 3 (2.3%) people changed due to gastrointestinal problems, such as diarrhea or indigestion. The remaining 8 (6.1%) patients changed because of the side effects of the existing medicine, and 10 (7.6%) changed due to unknown reasons. The ART types previously used by those in the switching group are listed in Table 2. 119 (90.8%) people used two types of nucleoside reverse transcriptase inhibitors (NRTIs) and integrase strand transfer inhibitors (INSTIs), among which the DTG-based 3DR was the most common, which was used by 101 (77.1%) people. Eight (6.1%) patients switched from bictegravir/emtricitabine/tenofovir alafenamide (BIC/FTC/TAF) to DTG/3TC.

### 3.2. Antiretroviral Resistance Test

We also performed an antiretroviral resistance test in each group which is shown in Table 3. This included 18 (90.0%) patients in the treatment-naïve group and 51 (38.9%) patients in the switching group. Five (25.0%) patients in the treatment-naïve group and 14 (10.7%) in the switching group were identified as being resistant to antiviral drugs. M184I mutations (low-level 3TC resistance) and H51HDNY mutations (low-level DTG resistance) were identified in the switching group. However, virological suppression was maintained even after the drug was changed to DTG/3TC. Other significant resistances were K101E (high-level rilpivirine resistance) in the treatment-naïve group, and K103N (high-level efavirenz and nevirapine resistance) and M230MI (intermediate-level nevirapine and rilpivirine resistance) in the switching group. These data are indicated in the Additional file.

### 3.3. Treatment Outcomes

#### 3.3.1. Effectiveness

**Figure 2:**
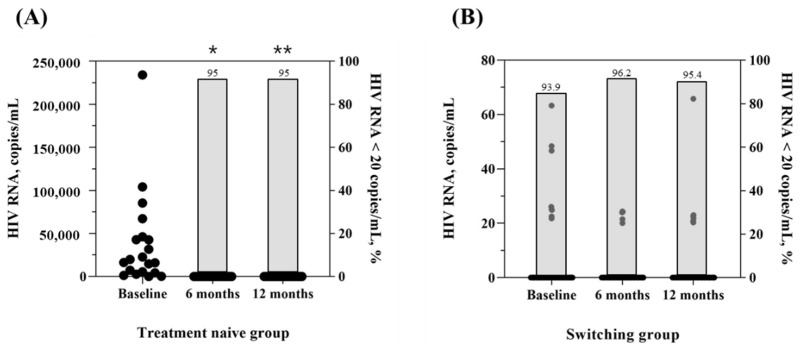
HIV RNA titer and proportion (%) of HIV RNA < 20 copies/mL in treatment naïve and switching group at baseline and 6- and 12-month follow-up after DTG/3TC treatment was started. (**A**) HIV RNA titer of the treatment-naïve group at baseline and 6- and 12-month follow-up; (**B**) HIV RNA titer of the switching group at baseline and 6- and 12-month follow-up. * *p* < 0.001 compared to the baseline. ** *p* < 0.001 compared to the baseline.

**Figure 3:**
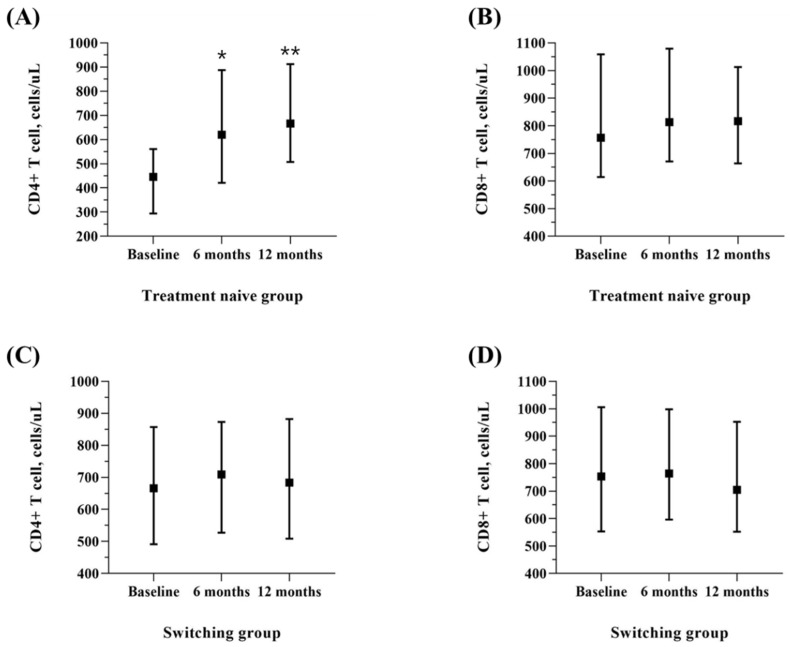
CD4+ and CD8+ T cell counts at baseline and 6- and 12-month follow-up after DTG/3TC treatment was started. (**A**) CD4 positive T cell counts of the treatment-naïve group at baseline and 6- and 12-month follow-up; (**B**) CD8 positive T cell counts of the treatment-naïve group at baseline and 6- and 12-month follow-up; (**C**) CD4 positive T cell counts of the switching group at baseline and 6- and 12-month follow-up; (**D**) CD8 positive T cell counts of the switching group at baseline and 6- and 12-month follow-up. * *p* < 0.001 compared to the baseline. ** *p* < 0.001 compared to the baseline.

As shown in Table 4 and Figure 2, the median HIV RNA titer before ART was 18,050.0 (4682.5, 45,350.0) copies/mL in the group treated with DTG/3TC as the initial therapy, and 0.0 (0.0, 0.0) copies/mL in the switching group, most of whom (93.9%) had a virologically suppressed status. HIV RNA <20 copies/mL was achieved in 95% of patients after 12 months who selected DTG/3TC as initial therapy. In the group switching to DTG/3TC, virologic suppression was maintained well after 6 and 12 months, and HIV RNA <20 copies/mL was achieved in 95.4% of patients after 12 months. The CD4 + T cell count increased from 445.5 cells/µL (294.3, 560.8) to 620.0 cells/µL (420.8, 887.5) after 6 months and 666.5 cells/µL (507.8, 912.3) after 12 months in the treatment-naïve group (*p* < 0.001). In the switching group, there was no significant difference in the CD4 + T cell count changes (*p* = 0.080) (Figure 3).

#### 3.3.2. Tolerability and Safety

As shown in Table 4, we evaluated profiles for lipids, such as triglycerides (TG), low-density lipoprotein (LDL), and cholesterol. There was no significant change before and after DTG/3TC administration, except for TG and cholesterol levels in the switching group. In the switching group, TG level changed from 188.91 ± 137.66 mg/dL to 180.19 ± 115.32 mg/dL at 6 months and to 161.52 ± 102.54 mg/dL at 12 months (*p* = 0.026). Cholesterol level also changed from 184.62 ± 38.64 mg/dL to 177.70 ± 37.47 mg/dL at 12 months (*p* = 0.004). There were no significant changes in blood urea nitrogen (BUN), aspartate aminotransferase (AST) and alanine aminotransferase (ALT), glucose control, or body weight before and after DTG/3TC administration in either group. But in both group, increase in creatinine observed from 0.9 mg/dL (0.8, 1.0) to 1.0 mg/dL (1.0, 1.1) and 1.1 mg/dL (1.0, 1.2) (*p* < 0.001) in treatment naïve and 0.99 ± 0.17 mg/dL to 1.04 ± 0.16 mg/dL and 1.02 ± 0.16 mg/dL (*p* < 0.001) respectively.

After starting DTG/3TC, five people (one treatment-naïve, four switching) complained of side effects. One treatment-naïve patient had an increase in AST up to three times the normal level for unknown reasons but continued to take the drug, and the AST level eventually returned to normal again. The remaining four patients complained of indigestion, diarrhea, constipation, and fatigue, but their symptoms were mild, and they were still taking the drug without changing the ART. None of the 151 patients who took DTG/3TC complained of serious side effects and were taking the drug without stopping.

## 4. Discussion

2DR based on DTG is recommended as a first-line therapy because of its high resistance barrier, low drug–drug interaction, and ease of administration [11,12]. In this study, we observed no virological failure in patients receiving DTG/3TC for both the treatment-naïve and treatment-switching groups. There were no serious side events including psychiatric adverse events [13], and none of the participants stopped taking the medication—these findings are consistent with other studies [5,14,15].

Previous clinical studies have not sufficiently included the Asian population. In the TANGO study, Asians accounted for only 3.5% of the total study population, and in the GEMINI study, Asians accounted for only 10% of the total study population [6,7]. In Asia, the number of PLWH in 2020 is estimated to be 5.8 million, which is approximately 15.4% of the global PLWH population. Considering the proportion of Asians worldwide, it is essential to study the effectiveness of DTG/3TC in Asians [16]. According to this study, the effectiveness of DTG/3TC was good in Asian patients. In addition, it could be used without any complications. Some studies have shown that second-generation INSTIs, such as DTG, increase body weight [17,18]; however, in our study, there were no major problems in body weight, lipid profile, or liver function until at least 12 months.

Nevertheless, it should be noted that mild deterioration of renal function was observed, although there was no case of worsening renal function defined as acute kidney injury (AKI) in the Kidney Disease Improving Global Outcomes (KDIGO) clinical practice guidelines [19]. The slight renal dysfunction observed is thought to be due to DTG because DTG inhibits tubular secretion of creatinine by inhibiting organic cation transporter 2 (OCT2), resulting in small, sustained, non-progressive increases in serum creatinine [20,21]. This has been demonstrated in several clinical studies [7,22,23,24]. Therefore, long-term follow-up for renal function is necessary.

This drug also showed positive results in patients with resistance mutations. For example, even in patients with M184I, a mutation with low-level resistance to 3TC, no viral blip or virological failure was observed. There have been no large-scale studies yet to prove the effect of 2DR including DTG/3TC in M184I, but several studies have shown the potential effect of 2DR based on DTG as a nucleoside-sparing regimen [25]. Further follow-up with the participants in our study, who are well-suppressed virologically, is planned.

This study has several limitations. We reviewed this as an observational study with fewer participants for limited days around 12 months and not a long-term follow-up, so virologic failure or some side effects may have been missing. In particular, several experts are concerned about complications associated with chronic exposure to ART drugs. Long-term exposure to integrase inhibitors, particularly DTG, persists significantly in neurocognitive impairment among some persons [26]. However, no one complained of any notable symptoms related to this during the 12-month period in this study. We only collected routine laboratory data; therefore, some data, such as bone metabolism marker assessment data, were missing in the analysis. And some studies reported the interaction of DTG/3TC with rifampicin, carbamazepine, and metformin. In particular, in the case of rifampicin, it could lower the DTG concentration when co-administered [27]. Conversely, metformin concentration may increase when taken together with DTG [28]. However, in this study, 20 tuberculosis patients, including latent tuberculosis, were identified, but none of them were taking rifampicin. Also, no one was taking carbamazepine, so we could not evaluate the drug-drug interaction with these drugs. After DTG/3TC administration, there were no patients who reduced their diabetes medication or had a significant change in their fasting serum glucose level. However, this study is to demonstrate the effects, effectiveness, and safety of DTG/3TC in an Asian population, and it may lay the foundation for recommending the use of 2DR based on DTG in Asian countries.

In conclusion, this study showed that DTG/3TC could be effectively used in patients without resulting in the occurrence of major adverse side effects. Therefore, DTG/3TC might be a good treatment option for PLWH in Korea.

## Figures and Tables

**Table 1:** Baseline characteristics of patients who treated with Dolutegravir/Lamivudine.

	Treatment Naive (N = 20)	Switching Group (N = 131)	*p*-Value
Gender, male	20 (100.0)	122 (93.1)	0.608
Age, years	28.0 (24.3, 41.3)	45.0 (35.0, 55.0)	<0.001
BMI, kg/m^2^	23.4 (22.3, 24.3)	23.7 (21.7, 25.9)	0.001
Sexual orientation *			
Heterosexual	4/13 (30.8)	30/82 (36.6)	0.765
MSM	6/13 (46.2)	44/82 (53.7)	0.615
Bisexual	2/13 (15.4)	5/82 (6.1)	0.244
Alcohol	13 (81.3)	59 (59.6)	0.097
Smoking	8 (47.1)	46 (44.2)	0.828
Initial CD4+ T cell, cell/uL	445.5 (294.3, 560.8)	665.0 (490.0, 858.0)	<0.001
Initial CD8+ T cell, cell/uL	756.5 (614.5, 1059.0)	746.0 (553.0, 1006.0)	0.308
Treatment duration of Dolutegravir/lamivudine, days	507.5 (421.5, 628.3)	525.0 (455.0, 550.0)	0.001
Resistance test	18 (90.0)	51 (38.9)	<0.001
Underlying disease			
Hypertension	2 (10.0)	30 (22.9)	0.189
Diabetes mellitus	2 (10.0)	25 (19.1)	0.531
Dyslipidemia	2 (10.0)	52 (40.0)	0.009
Cardiovascular disease	2 (10.0)	3 (2.3)	0.131
Osteoporosis	0 (0.0)	9 (6.9)	0.608
Osteopenia	3 (15.0)	20 (15.3)	0.999
Chronic kidney disease	1 (5.0)	9 (6.9)	0.999
Chronic lung disease	0 (0.0)	1 (0.8)	0.999
Tuberculosis	1 (5.0)	19 (14.5)	0.476
Syphilis	3 (15.0)	45 (34.6)	0.080
Solid tumor	1 (5.0)	3 (2.3)	0.437
Hematologic malignancy	0 (0.0)	4 (3.1)	0.999
Psychiatric illness	2 (10.0)	10 (7.6)	0.661
Hepatitis profile			
Hepatitis C virus antibody positivity	0 (0.0)	1 (0.8)	0.999
Recent diagnosed sexually transmitted disease	1 (5.0)	5 (3.8)	0.580

Data are presented as mean ± standard deviation or number (%) of patients, unless otherwise indicated. BMI; body mass index, MSM; Men who have sex with men. * sexual orientation: for the respondents, 13 people in the treatment naive and 82 in the switching group answered.

**Table 2:** Previously treated anti-retroviral therapy.

Latest Regimen	Latest ART	Number
2NRTIs + PIs	ABC/3TC/ATV/COBI	2
	3TC/ABC + ATV	1
	3TC/ABC + LPV/r	1
2NRTIs + INSTIs	DTG/ABC/3TC	101
	BIC/FTC/TAF	8
	ABC/3TC + RAL	5
	TDF/FTC + RAL	3
	TAF/FTC + RAL	1
	TAF/FTC + DTG	1
2NRTIs + NNRTIs	ETR + ABC + 3TC	1
	ZDV/3TC + EFV	2
	3TC/ABC + EFV	2
	TAF/FTC + EFV	1
NRTIs + PIs + INSTIs	EVG/COBI/FTC/TAF	2

Data are presented as number of patients. NRTIs; Nucleoside and nucleotide reverse transcriptase inhibitors, NNRTIs; Non-nucoleoside reverse transcriptase inhibitors, PIs; Protease inhibitors, INSTIs; Integrase strand trasnfer inhibitors, ABC; Abacavir, 3TC; Lamivudine, ATV; Atazanavir, COBI; Cobicistat, DRV; Darunavir, BIC; Bictegravir, FTC; Emtricitabine, TAF; Tenofovir alafenamide, TDF; Tenofovir disoproxil fumarate, RAL; Raltegravir, ETR; Etravirine, ZDV; Zodivudine, EFV; Efavirenz, EVG; Elvitegravir.

**Table 3:** Treatment resistance tests for the treatment-naïve and switching groups.

	Treatment Naive (N = 20)	Switching Group (N = 131)
Antiretroviral resistance test	18 (90.0)	51 (38.9)
Resistant to antiviral drugs	5 (25.0)	14 (10.7)
Major resistance type		
NRTIs	0 (0.0)	1 (0.7)
NNRTIs	4 (20.0)	11 (8.4)
INSTIs	2 (10.0)	3 (2.3)
PIs	0 (0.0)	0 (0.0)

Data are presented as number (%) of patients, unless otherwise indicated. NRTIs; nucleoside reverse transcriptase inhibitors, NNRTIs; non-nucleoside reverse transcriptase inhibitors, INSTIs; integrase strand-transfer inhibitors, PIs; protease inhibitors.

**Table 4:** Laboratory parameters at between baseline and 6 and 12 months for the treatment-naïve and switching groups.

Parameter	Treatment Naïve				Switching Group			
	Baseline	6 Months	12 Months	*p* Value ^+^	BASELINE	6 months	12 Months	*p* Value ^+^
HIV RNA, copies/mL	18050.0 (4682.5, 45350.0)	0.0 (0.0, 0.0)	0.0 (0.0, 0.0)	0.005 *	2.13 ± 9.14	0.88 ± 4.43	1.33 ± 7.05	0.252 *
CD4, cells/uL	445.5 (294.3, 560.8)	620.0 (420.8, 887.5)	666.5 (507.8, 912.3)	<0.001	692.50 ± 279.70	728.36 ± 293.71	703.66 ± 278.99	0.080
CD8, cells/uL	756.5 (614.5, 1059.0)	813.5 (671.3, 1079.3)	817.0 (664.0, 1013.0)	0.583	805.98 ± 348.62	841.16 ± 356.35	784.05 ± 347.54	0.007
Lipid profile								
Triglyceride, mg/dL	89.0 (71.5, 139.5)	126.5 (78.8, 159.3)	92.0 (78.0, 196.5)	0.135	188.91 ± 137.66	180.19 ± 115.32	161.52 ± 102.54	0.026 *
LDL, mg/dL	106.4 (81.1, 122.9)	104.1 (83.6, 129.6)	107.3 (89.2, 120.0)	0.993	109.70 ± 79.87	105.03 ± 31.75	103.96 ± 33.17	0.510 *
cholesterol, mg/dL	172.5 (158.8, 202.3)	178.0 (158.0, 212.8)	181.0 (160.1, 202.8)	0.719	184.62 ± 38.64	185.00 ± 36.56	177.70 ± 37.47	0.004
Renal profile								
blood urea nitrogen, mg/dL	12.0 (10.8, 15.2)	15.0 (11.8, 16.4)	14.7 (11.5, 15.8)	0.088	14.64 ± 3.75	15.23 ± 3.94	15.12 ± 3.82	0.177
creatinine, mg/dL	0.9 (0.8, 1.0)	1.0 (1.0, 1.1)	1.1 (1.0, 1.2)	<0.001	0.99 ± 0.17	1.04 ± 0.16	1.02 ± 0.16	<0.001 *
Liver function								
AST, IU/L	23.0 (17.5, 33.5)	20.0 (18.0, 30.0)	23.0 (17.0, 29.3)	0.342 *	25.89 ± 22.65	24.53 ± 13.26	26.14 ± 10.77	0.511 *
ALT, IU/L	26.5 (13.5, 32.3)	21.0 (16.0, 24.0)	21.0 (14.5, 35.0)	0.283 *	30.86 ± 30.23	29.28 ± 22.51	30.05 ± 20.32	0.726 *
Total bilirubin, mg/dL	0.8 (0.5, 1.0)	0.9 (0.7, 1.2)	0.8 (0.6, 1.0)	0.182	0.79 ± 0.40	0.89 ± 0.41	0.88 ± 0.35	0.003
Glucose, mg/dL	94.5 (90.5, 102.8)	93.0 (87.3, 94.8)	96.5 (89.0, 100.8)	0.361 *	104.85 ± 41.61	102.30 ± 19.11	105.05 ± 34.74	0.915
Body weight, kg	66.5 (62.3, 75.3)	70.0 (64.0, 78.0)	71.0 (64.0, 78.0)	0.070	70.0 (63.3, 78.8)	70.0 (63.0, 79.0)	70.0 (63.2, 78.8)	0.268 *

Data are presented as median (Interquartile range), unless otherwise indicated. LDL; low density lipoprotein, HDL; high density lipoprotein, AST; aspartate aminotransferase, ALT; alanine aminotransferase. * Using Greenhouse-Geisser correction. ^+^ Using repeated measure ANOVA.

## Data Availability

The original contributions presented in the study are included in the article. Further inquiries can be directed to the corresponding authors.
